# Infantile wryneck: report of 2 cases^☆^

**DOI:** 10.1016/j.bjorl.2016.05.011

**Published:** 2016-06-22

**Authors:** Jeyasakthy Saniasiaya, Irfan Mohamad, Siti Khairunnisaak Abdul Rahman

**Affiliations:** a Universiti Sains Malaysia Health Campus, School of Medical Sciences, Department of Otorhinolaryngology – Head & Neck Surgery, Kelantan, Malaysia; b Universiti Sains Malaysia Health Campus, School of Medical Sciences, Department of Radiology, Kelantan, Malaysia

## Introduction

Congenital wry neck following sternocleidomastoid (SCM) injury may lead to haematoma formation within the SCM or a condition known as fibromatosis colli.

SCM injury may occur following in-utero constraint, infection, structural or neurological deficit, neoplasm and trauma.^1^ Birth history of difficulties during delivery has been proposed by myriad authors as the main factor of this condition.

Ultrasonography (USG) of neck is the main modality for diagnosis. Albeit the usual spontaneous regression and favourable prognosis, this entity requires physiotherapy and follow-up as left untreated, may result in plagiocephaly, hemifacial hypoplasia and body distortion. We report two cases of torticollis secondary to SCM mass and their management. We would like to highlight that awareness of these entities are prudent as to avoid unnecessary and invasive investigations.

## Case 1

A 2 month-old child who was delivered via Low-Segment Caesarean-Section (LSCS) due to intrauterine breech presentation with prolonged labour presented with right neck swelling since birth. According to the mother, antenatal USG did not reveal any neck mass or any other abnormalities. The right neck swelling was noted to be gradually increasing in size for the past two months. Despite the increasing size of the mass, there were no accompanying obstructive symptoms and child was tolerating feeding well. The neck mass however, caused restricted movement of the neck on the affected side. Apart from that, there were no signs of an active infection and child was noted to be active by both the parents.

**Figure 1:**
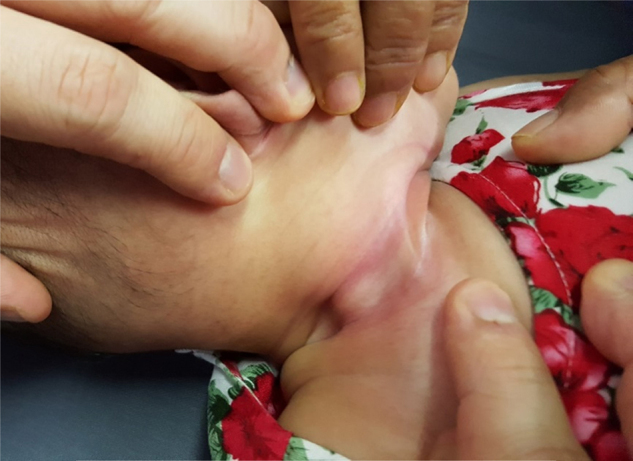
Right neck swelling measuring 3 cm × 3 cm extending over level II–III, firm to hard in consistency.

**Figure 2:**
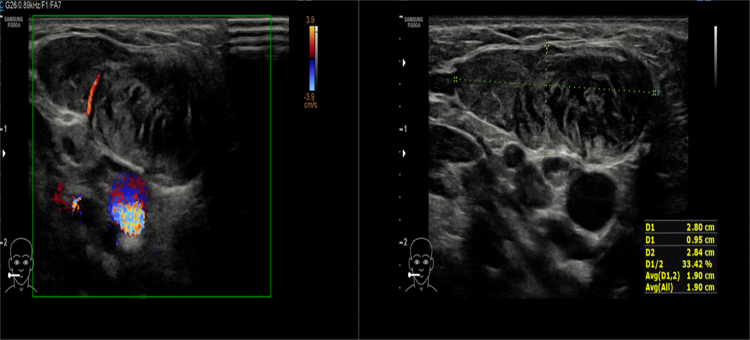
Longitudinal plane of right neck shows heterogenous hypoechoic lesion seen within the right SCM. This lesion measures 2.8 cm × 0.9 cm × 2.6 cm. No increased in colour Doppler seen within the lesion.

Clinically, child was active with no signs of respiratory distress. Upon examination of the neck mass, a right neck swelling measuring 3 cm × 3 cm extending over level II to III, firm to hard consistency, non-pulsatile, with no signs of inflammation was noted (Fig. 1). The overlying skin was not fixed to the mass. Limited range of neck movement was noted especially on the affected side. Other physical examination findings were unremarkable. Ultrasonography (USG) of neck revealed a heterogeneous solid lesion within the right SCM muscle measuring 2.8 cm × 0.9 cm × 2.6 cm with no increase in colour Doppler seen within the lesion. These features highly represent a right sternocleidomastoid haematoma (Fig. 2).

The infant was referred for physiotherapy and was given a 3 week appointment upon which the neck mass was noted to have tremendously subsided.

## Case 2

A 5 month-old child born to a primiparous mother, with a history of right sided neck swelling which has been slowly increasing in size for the past one month. The neck swelling was noted to be accompanied with limited movement of neck on the affected side. The parents claimed that no swelling was noted prior to that. The infant was delivered via Spontaneous Vaginal Delivery (SVD) after a prolonged labour period. There was no history of trauma or fall and child has been well and active otherwise. No signs of respiratory distress were noted by parents.

**Figure 3:**
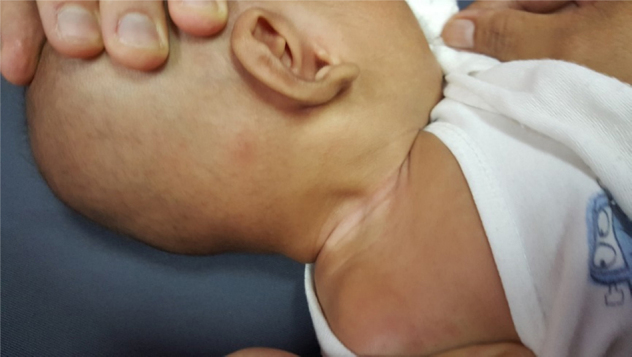
Right neck swelling measuring 2 cm × 3 cm, firm to hard consistency, non-pulsatile with no signs of active inflammation over the level II sternocleidomastoid region.

**Figure 4:**
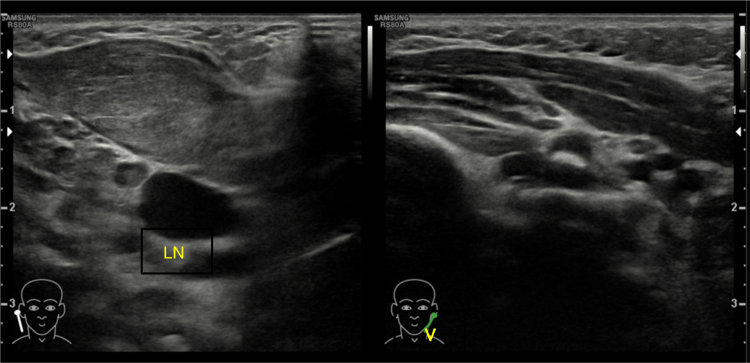
Right Sternocleidomastoid Muscle (SCM) is bulky as compared to the left with loss of normal striations. The lesion is anterior to the right common carotid artery and right internal jugular vein (V, internal jugular vein; LN, lymph node).

Physical examination revealed a right neck swelling measuring 2 cm × 3 cm, firm to hard consistency, non-pulsatile with no signs of active inflammation over the level II sternocleidomastoid region (Fig. 3). Torticollis was perceived on the affected side. USG neck performed demonstrated thickened right SCM in a fusiform manner, loss of the normal striae with homogenous increase in internal echogenicity, no hypervascularity or collection seen which was suggestive of fibromatosis colli (Fig. 4). The child was referred for physiotherapy and following a 3 course of physiotherapy within 6 weeks, the neck swelling and the torticollis completely resolved.

## Discussion

The term wryneck arises from old English word wrigan meaning to turn can also be defined as twisted or distorted.^2^ Congenital muscular torticollis may be of three forms: sternomastoid tumour or torticollis with a mass, torticollis without a mass and postural torticollis.^3^ Both of our patients belongs to the first group: sternomastoid tumour with a mass. The congenital muscular torticollis has preponderance of 0.4%.^3^

SCM tumour mostly occurs in infants born to primiparous mothers with nearly 60% of them with complicated delivery.^4^ Breech presentation is said to be the predominant factor leading to its occurrence^4^ regardless of the mode of delivery as foetal malposition is one of the main cause of congenital wryneck. Naturally, fibromatosis colli manifests 2 weeks after birth as a hard, immobile, fusiform swelling within the SCM which increases in size up to four weeks of life. It has predilection towards the left side of the neck.^5^ On the contrary, both of our patients has SCM mass on the right side.

Countless theories has been postulated regarding causative factor of wryneck, amongst which birth injury and ischaemia is the most sought out for.^6^ Birth trauma may lead to muscle stretching and haematoma formation due to the obstructed venous outflow during intrauterine development or during delivery^5^ which precipitates necrosis initially, then fibrosis followed by emergence of secondary pressure within muscle fibres causing SCM tumefaction.^7^ This may be the case for our 2nd infant discussed, whereby his primiparous mother underwent prolonged labour which would have contributed to the fibromatosis colli formation. However, fibromatosis colli may also be the underlying causative factor of torticollis even without an obvious neck mass. Lesion may also have taken place due to intra-uterine foetal head position resulting in selective injury to the SCM^8^ which may have led to a haematoma formation as in our 1st case.

SCM mass with congenital wryneck can be considered as a stigmata. General practitioners and paediatricians especially should not be confused with other neck masses over the SCM region including branchial cyst, hemangioma, lymphangioma, lipoma, sebaceous cyst, neuroblastoma, rhabdomyosarcoma, fibrosarcoma^9^ cystic hygroma, cervical lymphadenopathy, as this may delay the diagnosis and subsequent treatment.

USG is the best method to diagnose as it is safe, fast and non-invasive. Doppler ultrasonography can also be used to define high resistance waveform. On real time sonography, simultaneous movement between the mass and the rest of SCM is seen.^10^ Although, congenital wryneck with SCM tumour can be diagnosed via clinical examination, imaging studies may be needful especially to confirm the diagnosis and to rule out other conditions. As for fine-needle aspiration cytology, it may be mistaken for a fibrous neoplasia due to similar histological features.

Chen et al., reported that 90% of congenital torticollis resolves within a one-year period.^11^ Wryneck secondary to SCM mass is treated normally with physiotherapy which includes active and passive stretching of the SCM on the affected side. Physiotherapy is said to have 90–95% of success rate.^8^ As for surgical intervention it is reserved for patients with persistent SCM swelling after one year and for infants with craniofacial abnormalities.^12^ Common surgical methods includes; excision of SCM, bipolar release of SCM with Z-plasty reconstruction of muscle bulk^8^ and SCM tenotomy or release.

## Conclusion

Findings of torticollis in their little one's may cause any parent to become anxious, hence it is of dire importance that the physicians especially paediatricians become aware of such entity and its management. As delaying appropriate physiotherapy may cause devastating outcomes and unnecessary burden both parents and physicians.

## Conflicts of interest

The authors declare no conflicts of interest.
